# Clinical Course of Mechanically Ventilated COVID-19 Patients With Pneumothoraces

**DOI:** 10.7759/cureus.16704

**Published:** 2021-07-28

**Authors:** Taha Mallick, Max Murray Ramcharan, Anant Dinesh, Mahera Hasan, Ryan Engdahl, Alexius Ramcharan

**Affiliations:** 1 Surgery, Harlem Hospital Center, New York, USA; 2 General Surgery, Harlem Hospital Center, New York, USA; 3 Transplant Surgery, University of Minnesota, New York, USA; 4 Surgery, Dow University of Health Sciences, Dow International Medical College, Karachi, PAK

**Keywords:** covid-19, pneumothorax, invasive mechanical ventilation, clinical course, mortality rate in covid-19

## Abstract

Introduction

Pneumothoraces in mechanically ventilated patients with COVID-19 indicate severe lung damage from inflammatory injury and barotrauma. These patients have a high mortality rate, and additional factors may further alter their clinical course.

Methods

We conducted a retrospective review of patients admitted to 11 public hospitals in New York City between March 6 and April 9, 2020, diagnosed with COVID-19. We identified 39 patients who developed pneumothoraces immediately after intubation or after a period of time on mechanical ventilation. Our study population was divided into various groups using demographic and clinical characteristics. Statistical analyses were conducted using SPSS software (IBM Inc., Armonk, USA) and paired t-tests to compare clinical outcomes between the various groups. P values < 0.05 were considered statistically significant.

Results

Our population was comprised of 28 male (72%) and 11 female patients; 36 out of 39 patients (92.3%) died with a median time of 10 days from admission to death and a median time of 2 days from pneumothorax to death. The remaining three were discharged home or to another facility. Pneumothoraces developed immediately after intubation in 18 patients and after a period of time on mechanical ventilation in 21 patients. Factors associated with a worse clinical course included age greater than 65 years (time from admission to pneumothorax 4.81 vs 8.35 days; p = 0.011) and presence of one or more comorbidities (time from admission to intubation 2.3 days vs 4.8 days; p = 0.041). Other factors that may worsen clinical course include previous smoking (time from admission to pneumothorax 4.4 vs 8.54 days; p = 0.074) and use of positive end-expiratory pressure (PEEP) greater than 15 cm H_2_O (time from intubation to pneumothorax 3.89 vs 6.42 days; p = 0.14).

Conclusions

Based on the findings in our retrospective review, COVID-19 patients who develop pneumothoraces on mechanical ventilation have a mortality rate in excess of 90%. Older patients and those with comorbidities have a more fulminant clinical course.

## Introduction

COVID-19 pneumonia is associated with alveolar damage [1] and microvascular thrombosis [2] leading to varying degrees of respiratory compromise ranging from a mild respiratory illness to respiratory failure with acute respiratory distress syndrome (ARDS). In this study, we focus on COVID-19 patients who require intubation and mechanical ventilation and develop pneumothoraces. We find that these patients have very high mortality and aim to analyze certain patterns and trends in their demographic characteristics and clinical course. Previous experience with the severe acute respiratory syndrome (SARS) pandemic left us with important lessons regarding the pathogenesis of pneumothorax in those patients, which may involve inflammatory injury to lung parenchyma [3]. The SARS-CoV-2 belongs to the same family of viruses, and it seems likely that the mechanism of development of pneumothorax in SARS and COVID-19 patients would be similar. Therefore in this study, we also study levels of various inflammatory markers indicating the severity of COVID-19 in our patients who develop pneumothoraces.

## Materials and methods

This study was planned as a retrospective review of mechanically ventilated COVID-19 patients who develop pneumothoraces. IRB approval was obtained prior to data collection.

Using Epic software (Epic Systems Corporation, Wisconsin, United States), we conducted a retrospective review of 5412 consecutive patients admitted between March 6 and April 9, 2020, who were diagnosed with COVID-19 based on reverse transcription-polymerase chain reaction (RT-PCR) testing of nasopharyngeal swab specimens. Among these patients, 64 were found to have a concomitant diagnosis of pneumothorax. We then proceeded with manual chart checking and identified 39 patients who developed a pneumothorax either immediately after intubation or after a period of time on mechanical ventilation. We excluded patients with pneumothoraces attributable to central line insertion or malpositioned nasogastric tubes, those with spontaneous pneumothoraces (those not requiring intubation and mechanical ventilation), and those with pneumothorax in a past admission.

We divided our patients into two groups: (1) those with post-intubation pneumothoraces (group 1 comprised of 18 patients of which 14 developed pneumothoraces after their first intubation, while four developed pneumothoraces after re-intubation) and (2) those who developed pneumothoraces after a period of time on mechanical ventilation (group 2 comprised of 21 patients).

For each group, we first studied demographic characteristics including age, gender, and BMI. We also studied the presence of previous medical conditions and their smoking histories.

For each group, we evaluated the clinical course to compute the following parameters: time from admission to intubation, time from intubation to pneumothorax (which was considered zero in case of post-intubation pneumothoraces), and time from admission to pneumothorax. For patients who died in hospital, we also calculated the time from admission to death and time from pneumothorax to death.

In addition, for each group, we calculated the mean of their maximum white blood cell counts, maximum neutrophil counts, minimum lymphocyte counts, maximum C-reactive protein levels, maximum lactate dehydrogenase levels, and maximum ferritin levels. We then conducted a comparison of these values between the two groups looking for any significant differences. All lab values were rounded to three significant figures.

We also subdivided our study population based on age, gender, BMI, smoking status, and previous comorbidities and studied differences in the above-mentioned clinical parameters between the various groups. Finally, we analyzed differences in the clinical course of patients based on the maximum PEEP used prior to the development of pneumothorax.

## Results

Fourteen patients developed pneumothoraces immediately after intubation, four developed pneumothoraces after re-intubation, and 21 developed pneumothoraces after a period of time on positive pressure ventilation. The median age of our patients was 60 years with an age range of 26-82 years. Twenty-eight patients (72%) were male. Thirty-six patients (92%) were deceased at the time of this review. Two patients had been discharged from the hospital of which one has been seen as an outpatient and is doing well, while the other was discharged to a skilled nursing facility with no further information available. One patient was sent to an outside facility for extracorporeal membrane oxygenation (ECMO) therapy, and further information on outcomes was not available.

Findings in both groups

The median age of our patients was 60 years with an age range of 26-87 years. Median BMI was 29.5 kg/m^2^ with a range from 19.5 to 56.1 kg/m^2^ (BMI was not available for two patients).

No patients were reported as active smokers in the electronic medical records (EMR). Twenty-four patients were reported as non-smokers, five were reported as former smokers, and smoking status was unknown for 10 patients.

Table 1 shows the associated comorbidities in our patient population.

**Table 1:** Frequency of comorbid medical conditions in our patient population COPD, Chronic obstructive pulmonary disease.

Disease	None	Diabetes mellitus	Hypertension	Pulmonary disease (COPD, asthma)	Cardiac disease	Chronic kidney disease	Chronic liver disease	Endocrine disease	Cancer	Autoimmune disease	Other
Frequency	17	15	17	5	4	1	1	1	2	0	4 (diverticulitis, gallstone pancreatitis, dyslipidemia)

The median time from admission to intubation was three days (interquartile range 0-5 days), and the median time from admission to pneumothorax was six days (interquartile range 3-10 days).

For patients who died (36 out of 39 patients), the median time from admission to death was 10 days (interquartile range 7-13 days), and the median time from development of pneumothorax to death was two days (interquartile range 1-5 days).

The mean maximum white blood cell count was 24.1 x 103/ul, mean maximum neutrophil count was 20.5 x 103/ul, mean minimum lymphocyte count was 0.525 x 103/ul, mean maximum C-reactive protein level was 198 mg/L, mean maximum lactate dehydrogenase level was 934 U/L, and mean maximum ferritin level was 2680 ng/ml. 

Findings in Group 1

The median age was 62.5 years with an age range of 27-87 years. Median BMI was 30.2 kg/m^2^ with the range from 22.7 to 56.1 kg/m^2^ (BMI was not available for two patients). 

No patients were reported as active smokers in the EMR. Eight were reported as non-smokers, three were former smokers, while smoking status was unknown for seven patients.

The median time from admission to intubation was 3.5 days (interquartile range 0.25-6.75 days), and the median time from admission to pneumothorax was 4.5 days (interquartile range 1.5-7.75 days). The time from intubation to pneumothorax in these patients was considered zero days (since these were post-intubation pneumothoraces). The time from admission to pneumothorax was the same as the time from admission to intubation except for the four patients who developed pneumothoraces after re-intubation.

For patients who died (17 out of 18 patients), the median time from admission to death was 10 days (interquartile range 7-12 days), and the median time from development of pneumothorax to death was two days (interquartile range 1-4 days). 

For these patients, the mean maximum white blood cell count was 20.1 x 103/ul, mean maximum neutrophil count was 17.3 x 103/ul, mean minimum lymphocyte count was 0.596 x 103/ul, mean maximum C-reactive protein level was 136 mg/L, mean maximum lactate dehydrogenase level was 978 U/L, and mean maximum ferritin level was 1870 ng/ml. 

Findings in group 2

The median age was 59 years with an age range of 26-82 years. Median BMI was 27.1 kg/m^2^ with a range of 19.5-39.3 kg/m^2^.

No patients were reported as active smokers in the EMR. Sixteen patients were reported as non-smokers, two were reported as former smokers, while smoking status was unknown for three patients.

The median time from admission to intubation was two days (interquartile range 0-4 days), the median time from intubation to pneumothorax was four days (interquartile range 2-10 days), and the median time from admission to pneumothorax was seven days (interquartile range 6-12 days).

For patients who died (19 out of 21 patients), the median time from admission to death was 11 days (interquartile range 8-16.5 days), and the median time from development of pneumothorax to death was two days (interquartile range 1-4.5 days). 

For these patients, the mean maximum white blood cell count was 27.5 x 103/ul, mean maximum neutrophil count was 23.2 x 103/ul, mean minimum lymphocyte count was 0.465 x 103/ul, mean maximum C-reactive protein level was 252 mg/L, mean maximum lactate dehydrogenase level was 889 U/L, and mean maximum ferritin level was 3370 ng/ml.

Group 2 was further divided into patients with maximum positive end-expiratory pressure (PEEP) of less than 15 cm H_2_O (group 2A; n = 9) and patients who had a maximum PEEP of 15 cm H_2_O or greater (group 2B; n = 12) prior to the development of pneumothorax. The meantime for the development of pneumothorax from intubation was found to be longer in group 2A than group 2B although this finding did not reach statistical significance (6.42 days vs 3.89 days; p = 0.14). A similar trend was noted in the time from pneumothorax to death for groups 2A and 2B (6.18 days vs 2.13 days; p = 0.185).

Analysis of findings

Differences in Clinical Course Between Groups 1 and 2

Significant differences were noted between the two groups in the meantime from admission to pneumothorax (4.94 vs 8.57 days; p = 0.014) and admission to death (8.65 vs 13.0; p = 0.028). No significant difference was noted in the time from admission to intubation (3.61 vs 3.24 days; p = 0.742) or time from development of pneumothorax to death (3.41 vs 4.47; p = 0.556).

Trends in Inflammatory Markers

Our patient population shows elevations in white blood cell and neutrophil counts, C-reactive protein (CRP), and lactate dehydrogenase (LDH) levels as well as lymphopenia. These factors have been associated with high mortality in COVID-19 patients, which is consistent with the results of our study.

On comparison of lab values, significant differences were noted between the two groups in the mean maximum white blood cell count (20.1 vs 27.5; p = 0.013), mean maximum neutrophil count (17.3 vs 23.2; p = 0.01), and mean maximum C-reactive protein level (136 vs 252; p = 0.01). The mean minimum lymphocyte count was lower in group 2, but this difference did not reach statistical significance (0.596 vs 0.465; p = 0.105). No significant difference was found in the mean maximum lactate dehydrogenase level (978 vs 889; p = 0.655) and mean maximum ferritin level (1870 vs 3370; p = 0.301).

Difference in Survival Trends Based on Age, Gender, and BMI

Compared to patients greater than or equal to 65 years of age, patients younger than age 65 years had a long time from admission to intubation (3.87 vs 2.75 days; p = 0.326). The time from admission to development of pneumothorax (8.35 vs 4.81 days; p = 0.018) was significantly higher for patients younger than age 65 years. Patients younger than age 65 years also had a long time from admission to death (12.4 vs 9.19 days; p = 0.118) although again this result did not reach statistical significance. The time from pneumothorax to death was 3.65 days for patients younger than 65 years and 3.38 days for those who are 65 years or older (p = 0.69).

No significant differences were found between male and female patients in the meantime from admission to intubation (3.71 vs 2.64; p = 0.388), admission to pneumothorax (7.64 vs 5.00; p = 0.114), or admission to death (10.8 vs 11.4; p = 0.786). The time from pneumothorax to death was shorter in males but again did not reach statistical significance (2.92 vs 6.36; p = 0.072).

Patients with BMI less than 35 kg/m^2^ were compared to those with BMI 35 or higher. No significant differences were noted in time from admission to intubation (3.61 vs 3 days; p = 0.659), admission to pneumothorax (7.54 vs 5.89 days; p = 0.366), admission to death (11.4 vs 11.6 days; p = 0.933), or pneumothorax to death (3.64 vs 5.67 days; p = 0.341).

Difference in Survival Trends Based on Smoking Status

As mentioned in the results section, no patients were reported as active smokers in the EMR on review. However, an analysis conducted to compare patients reported as non-smokers with those reported as former smokers yielded interesting results. Patients who had never smoked had a longer time from admission to intubation (4.13 vs 1.8 days; p = 0.186), admission to pneumothorax (8.54 vs 4.4 days; p = 0.074), and admission to death (12.8 vs 9 days; p = 0.229). The time from pneumothorax to death for non-smokers was 4.27 days and for former smokers was 4.6 days (p = 0.911).

Trends Based on Patient Comorbidities

Twenty-two out of 39 patients (56.4%) had at least one comorbidity. The most common comorbidity was hypertension (17 out of 39 patients, 43.6%) followed by diabetes mellitus (15 out of 39 patients, 38.5%). Five out of 39 patients had a history of COPD (12.8%). When compared to patients with one or more comorbidities, the meantime from admission to intubation was significantly longer for patients without comorbidities (4.82 vs 2.32 days; p = 0.041). Patients without comorbidities also had a long time from admission to pneumothorax (8.41 vs 5.72 days; p = 0.076) and admission to death (12.7 vs 9.82 days; p = 0.162) although these values did not reach statistical significance. The time from pneumothorax to death was 3.79 days for patients without comorbidities and 4.09 days for patients with one or more comorbidities.

Trends Based on Ventilator Settings

Group 2 was further divided into patients with a maximum PEEP of less than 15 cm H_2_O (group 2A) and patients who had a maximum PEEP of 15 cm H_2_O or greater (group 2B) prior to the development of pneumothorax. The meantime to development of pneumothorax from intubation was found to be longer in group 2A than group 2B although this finding did not reach statistical significance (6.42 days vs 3.89 days; p = 0.14). A similar trend was noted in the time from pneumothorax to death for groups 2A and 2B (6.18 days vs 2.13 days; p = 0.185).

The important findings in our study are summarized in Table 2.

**Table 2:** Summary of important findings in the clinical course of our patients

Patient characteristics		Meantime from admission to intubation (days)	Meantime from admission to pneumothorax (days)	Meantime from admission to death (days)	Meantime from pneumothorax to death (days)
Age	< 65 years (n = 23)	3.87	8.35	12.4	3.65
	≥ 65 years (n = 16)	2.75	4.81	9.19	4.38
	p-value	p = 0.326	p = 0.011	p = 0.118	p = 0.690
Gender	Male (n = 28)	3.71	7.64	10.8	2.92
	Female (n = 11)	2.64	5.00	11.4	6.36
	p-value	p = 0.388	p = 0.114	p = 0.786	p = 0.172
BMI	≥ 35 kg/m^2^ (n = 9)	3.61	7.54	11.4	3.64
	< 35 kg/m^2^ (n = 28)	3.00	5.89	11.6	5.67
	p-value	p = 0.659	p = 0.366	p = 0.933	p = 0.341
Smoking history	Negative (n = 24)	1.80	4.40	9.00	4.60
	Positive (n = 5)	4.13	8.54	12.8	4.27
	p-value	p = 0.186	p = 0.074	p = 0.229	p = 0.911
Comorbidities	None (n = 17)	4.82	8.41	12.7	3.79
	1 or more (n = 22)	2.32	5.72	9.82	4.09
	p-value	p = 0.041	p = 0.076	p = 0.162	p = 0.869

## Discussion

Etiology of pneumothorax in mechanically ventilated COVID-19 patients

Pathologic findings in the lungs of patients with COVID-19 are predominantly those of diffuse alveolar damage and airway inflammation [1] along with a possible role for pulmonary microvascular thrombosis [2]. Our patients mentioned above have extremely high mortality (92.3%) with 75% of deaths occurring five days after the development of pneumothorax even with pleural cavity decompression. This may indicate the severity of the underlying disease process in mechanically ventilated COVID-19 patients who develop pneumothoraces.

There have been case reports of COVID-19 patients with pneumothoraces, pneumomediastinum, and giant lung bullae even without the use of invasive mechanical ventilation indicating that barotrauma may not be the only pathogenic factor involved in pneumothorax development [4]. An article based on spontaneous pneumothoraces in SARS patients indicated that the underlying mechanism may involve inflammation-mediated pulmonary parenchymal injury, cystic changes, and then pneumothorax or pneumomediastinum [3]. Since SARS-CoV-2 belongs to the same coronavirus family as the SARS virus, it is plausible that they have similar pathogenetic mechanisms.

Several case reports of COVID-19 patients with spontaneous pneumothorax are available in the literature where patients eventually recover and are discharged from the hospital [4,5]. This is in contrast to our group of patients with pneumothorax while on mechanical ventilation who tend to have a much poorer prognosis with a very high mortality rate of 92.3%. This rate reflects the high mortality rates seen in other studies evaluating critically ill COVID-19 patients [6,7]. A similar trend was noted in SARS patients with pneumothoraces on mechanical ventilation [8].

The currently available literature indicates that higher white blood cell (WBC) count, neutrophil count, and inflammatory markers as well as lower lymphocyte counts are associated with more severe disease [9]. The mean peak WBC count; mean peak neutrophil count; and mean peak CRP, LDH, and ferritin levels in our patients are noted to meet the criteria mentioned in this paper [9] indicating that pneumothorax in patients with COVID-19 tends to occur in those with more severe disease. The authors, therefore, believe that pneumothoraces in ventilated COVID-19 patients arise by a combination of the mechanism described in the SARS paper [3] and barotrauma from mechanical ventilation.

Based on the findings in our study, the development of pneumothorax in patients on mechanical ventilation indicates a very poor prognosis probably due to the severity of the underlying lung disease. A recent article described outcomes in 60 patients with pneumothoraces with a much higher survival rate although it must be noted that this study included both ventilated and non-ventilated patients across multiple centers [10].

Pleural cavity decompression may prevent rapid progression to tension pneumothorax but probably does not alter the overall prognosis, which is related to pulmonary parenchymal injury and microvascular thrombosis. Thoracoscopic management of pneumothorax with bleb resection in patients on mechanical ventilation has also been described although final patient outcomes with this approach remain unclear [11]. 

Clinical course of mechanically ventilated COVID-19 patients with pneumothorax

Several points are notable about the clinical course of the patients involved in this study. Not only do these patients have a very high mortality rate (92.3%), they also seem to show rapid progression to death once pneumothorax develops. Of patients who died, the median time from admission to death was 10 days (interquartile range 7-13 days), and the median time from development of pneumothorax to death was two days (interquartile range 1-5 days). This indicates that 75% of patients who developed a pneumothorax died within five days. 

There also appears to be some difference in the clinical course between patients in group 1 (post-intubation pneumothorax) and group 2 (pneumothorax after a period of time on mechanical ventilation). Significant differences were noted between groups 1 and 2 in the meantime from admission to pneumothorax (4.94 vs 8.57 days; p = 0.014) and admission to death (8.65 vs 13.0 days; p = 0.028). No significant difference was noted in the time from admission to intubation (3.61 vs 3.24 days; p = 0.742) or time from development of pneumothorax to death (3.41 vs 4.47 days; p = 0.556). This indicates that patients in group 1 have a more rapid decline; but once pneumothorax develops, survival times between the two groups become similar.

Trends in inflammatory markers

Interestingly, on comparison between groups 1 and 2, it is noted that patients in group 2 have significantly higher mean maximum WBC count, neutrophil count, and CRP level compared to patients in group 1. Patients in group 2 also have a lower mean minimum lymphocyte count. However, despite these findings, patients in group 1 seem to have a more rapid clinical decline as noted above.

Difference in survival trends based on age, gender, and BMI

In our study population, we note that patients younger than age 65 years have a longer time from admission to pneumothorax and admission to death, the former of which reaches a statistical significance while the latter does not. However, once a pneumothorax develops again, there is no significant difference between the two groups in the time to death. A decrease in survival with advanced age in COVID-19 patients with pneumothoraces has also been noted in the article mentioned above [10] as well as studies from Lombardy, Italy [6]; Michigan, USA [12]; and the Bronx, New York [13]. 

In the comparison between male and female patients, no significant differences are noted in the clinical course, but the time from the development of pneumothorax to death is found to be higher in females. This difference is noted to approach but does not reach statistical significance indicating that in a larger sample of patients, it may be noted that female patients are able to survive longer once pneumothorax develops. Other studies in the literature do show decreased survival in male patients [6,13].

Finally, in our study population, no difference in the clinical course was noted between patients with BMI less than 35 and those with BMI 35 or higher. However, data from Bronx, New York, has shown an increased risk of mortality in patients with BMI 35 kg/m^2^ or higher [13]. Another meta-analysis has shown an increased risk of severe COVID, ICU admission, intubation, and mortality with obesity [13,14]. Obesity has also been shown to be associated with increased mortality in patients younger than 50 years [15]. Due to the very high mortality noted in our study population, it may not be possible to differentiate the impact of obesity using mortality rates; but with larger sample size, differences in the clinical course may become apparent.

Difference in survival trends based on smoking status

Since none of our patients were recorded as active smokers, we proceeded to compare patients recorded as former smokers with patients recorded as never smokers. Patients who had never smoked appeared to have longer times from admission to intubation, admission to pneumothorax, and admission to death although these results did not reach statistical significance. This trend may indicate that previous smoking history can worsen the clinical course in patients who match our study population. Smoking is known to be associated with increased mortality in COVID-19 patients in other studies [16]. One mechanism may involve increased lung inflammation and damage [17].

Differences in survival based on patient comorbidities

Studies have shown an increased risk of mortality in COVID-19 patients with an increasing number and severity of comorbidities [6,12,13]. Our study shows that 53.6% of patients had at least one comorbidity, which was similar to findings in other study populations [6,12,13].

Compared to patients with no comorbidities, patients who had at least one comorbidity had a significantly lower time from admission to intubation (2.3 days vs 4.8 days; p = 0.041). They also had a lower time from admission to pneumothorax (5.7 days vs 8.4 days; p = 0.076) and admission to death (9.8 days vs 12.7 days; p = 0.162) although these findings did not reach statistical significance. The times from pneumothorax to death were similar between patients with no comorbidities (3.8 days) and at least one comorbidity (4.1 days).

Effect of ventilator settings

Pneumothorax in patients with COVID-19 is believed to arise from a combination of barotrauma, inflammation, and microvascular thrombosis [1,2,11]. In our study, patients who required a PEEP of less than 15 did show longer survival times, a finding that may have reached a statistical significance in a larger study population. Data from Lombardy [6] indicates that the use of higher PEEP is associated with higher mortality. 

Limitations of our study

The authors recognize several limitations of this study, the most important being the fact that our patient sample is a dynamically changing one and more patients from the initial sample of 5412 may develop pneumothoraces with the passage of time. Therefore, this study would not be helpful in predicting the incidence of pneumothoraces in patients hospitalized with COVID-19. However, the sample of 39 patients generated by us has been studied to completion (all patients were either deceased or discharged from hospital at the end of our analysis except one patient who was transferred to an outside hospital for ECMO on whom information on the complete clinical course could not be obtained). The authors, thus, believe this study to be useful in helping providers to better predict and anticipate the clinical course of patients who fit the criteria of our study sample. 

Another limitation lies in the completeness of the availability of laboratory data. As indicated below, the levels of inflammatory markers were not uniformly available in all patients. In addition, in some cases even when lab values were available, a numerical value was not provided (e.g., C-reactive protein levels recorded as > 300 mg/L).

## Conclusions

COVID-19 patients who develop pneumothoraces on mechanical ventilation have mortality in excess of 90%. Older patients and patients with comorbidities have more fulminant clinical courses.
